# Measuring the Effect of Housing Quality Interventions: The Case of the New Zealand “Rental Warrant of Fitness”

**DOI:** 10.3390/ijerph14111352

**Published:** 2017-11-07

**Authors:** Lucy Telfar-Barnard, Julie Bennett, Philippa Howden-Chapman, David E. Jacobs, David Ormandy, Matthew Cutler-Welsh, Nicholas Preval, Michael G. Baker, Michael Keall

**Affiliations:** 1 Department of Public Health, University of Otago, Wellington 6021, New Zealand; julie.bennett@otago.ac.nz (J.B.); philippa.howden-chapman@otago.ac.nz (P.H.-C.); nicholas.preval@otago.ac.nz (N.P.); michael.baker@otago.ac.nz (M.G.B.); michael.keall@otago.ac.nz (M.K.); 2 School of Public Health, University of Illinois at Chicago, Chicago, IL 60612, USA; dejacobsdc@gmail.com; 3 National Center for Healthy Housing, Columbia, MD 21044, USA; 4 Warwick Medical School—Health Sciences, University of Warwick, Coventry CV5 6EG, UK; david.ormandy@warwick.ac.uk; 5 Home Style Green, Auckland 1072, New Zealand; matthew@homestylegreen.com

**Keywords:** housing quality, tenure, policy, evaluation, standards, health, economic, housing standards, housing assessment, home health and safety

## Abstract

In New Zealand, as in many other countries, housing in the private-rental sector is in worse condition than in the owner-occupier housing sector. New Zealand residential buildings have no inspection regime after original construction signoff. Laws and regulations mandating standards for existing residential housing are outdated and spread over a range of instruments. Policies to improve standards in existing housing have been notoriously difficult to implement. In this methods paper, we describe the development and implementation of a rental Warrant of Fitness (WoF) intended to address these problems. Dwellings must pass each of 29 criteria for habitability, insulation, heating, ventilation, safety, amenities, and basic structural soundness to reach the WoF minimum standard. The WoF’s development was based on two decades of research on the impact of housing quality on health and wellbeing, and strongly influenced by the UK Housing Health and Safety Rating System and US federal government housing standards. Criteria were field-tested across a range of dwelling types and sizes, cities, and climate zones. The implementation stage of our WoF research consists of a non-random controlled quasi-experimental study in which we work with two city-level local government councils to implement the rental WoF, recruiting adjoining council areas as controls, and measuring changes in health, economic, and social outcomes.

## 1. Introduction

Housing and the home are well-recognised as environmental and social determinants of health, with housing quality shown to be associated with mental, physical, and general health status [1]. Improvements to cold indoor temperatures improve respiratory health in children [2,3] and prevent mortality among elderly with circulatory disease [4], and the removal of home safety hazards reduces injury rates [5,6].

Rental housing is generally in worse condition than owner-occupied housing due to a combination of split incentives [7,8] and, presumably, insufficient or absent rental housing quality regulation and enforcement, though these latter topics are under-researched. With the economically vulnerable more likely to live in rental housing, the health risks associated with poor housing contribute to, and are compounded by, broader health inequalities. Meanwhile, landlords’ financial capital provides political capital, making it a long and complex process to improve policy on rental housing standards.

Since housing is a health determinant, housing policy is also in part health policy. Good policy is ideally informed by evidence, not just of the association between environmental exposure and outcome, but also of policy intervention and outcome.

This methods paper describes the background, development, and proposed implementation of a New Zealand (NZ) rental housing standard, the Rental Housing Warrant of Fitness (WoF), built on research evidence and informed by collaborators’ experiences with the UK Housing Health and Safety Rating System (HHSRS) and several enforcement approaches in the US. We describe the design of a new policy experiment that aims to measure outcomes for health alongside effects on rents or rental supply to provide a basis for a national rollout of a rental WoF. The effects of the WoF’s implementation will be measured within a health intervention framework.

## 2. The New Zealand Context

In NZ’s 2013 census, thirty-nine percent (39%) of homes were held in rental tenure, the highest level since 1951 and a marked increase from the low of 23% of households in rental tenure in 1991. Rentals house nearly half the population. Rental tenure is skewed by socio-economic status to such an extent that tenure is included as a variable in the production of NZ’s most widely used measure of socio-economic deprivation (NZDep) [9]. Over 70% of children in poverty live in rental housing [10]. Rental tenure is also more likely where the head of household is a single parent, of Māori or Pacific ethnicity, and/or when children are younger.

People in the lowest income quartile pay more than half their income in housing costs [11]. Lower income tenants have fewer rental options because they have low rental budgets and are more likely to have poor credit records and/or to belong to social and cultural groups commonly subject to discrimination or unconscious bias from landlords [12,13]. Their limited options do not include the luxury of choosing one of the scarce examples of good quality housing available in the rental market; even if they could pay more, a higher rent is no guarantee of a warm, dry house. 

### 2.1. NZ Housing Quality

NZ housing has well recognised quality problems that affect the health and safety of occupants. High seismic risk and the availability of locally grown timber led to widespread use of timber framing and cladding. Insulation requirements were only introduced in 1978, and low housing renewal rates have left NZ housing with low insulation levels and little thermal mass. Indoor temperatures seldom reach WHO recommended minima [14,15]. Building research and industry surveys have found mould, poor maintenance, and insufficient insulation and ventilation to be widespread [16,17]. These surveys, and other research [18], have also found NZ rental housing to be in worse condition than owner-occupied homes.

### 2.2. NZ Housing Quality Regulation

Most of NZ’s few laws and regulations on housing quality are dispersed over three main instruments. The first of these is the 2008 Building Code. Some of its standards, such as airtightness, ventilation, or installed heating, do not have performance measurement requirements. It requires insulation but not heating. Local authorities enforce it well in new dwellings, but buildings are only inspected and required to meet Code standards at the time of construction and do not need to be maintained to that standard after sign-off. Existing residential buildings are not required to be brought up to Code standard unless they are reported and assessed as insanitary or imminently dangerous. There is also evidence that even the Code’s low minimum standards for measures such as insulation are not met in most real-world instances [19]. 

The second instrument, the Housing Improvement Regulations 1947 (HIR), lays out some minimum standards for housing, but is outdated: a fireplace and chimney is assumed to be the default form of heating, there are references to privies, and there is no interpretation advice for studio units or other spaces where boundaries between bedroom, living space, and kitchen are blurred. The HIR are also of little effect due to lack of enforcement by local authorities or the Tenancy Tribunal [20].

The third instrument is the Residential Tenancies Act 1986 (RTA). The RTA requires landlords to provide premises in a reasonable state of cleanliness and in “a reasonable state of repair having regard to the age and character of the premises and the period during which the premises are likely to remain habitable and available for residential purposes”, to have smoke alarms, and, from July 2019, to have ceiling and underfloor insulation to a standard set somewhat below the 2008 Building Code (properties with existing insulation that meets 1978 Building Code regulations do not need to upgrade to 2008 Building Code standard, and properties with ceiling or underfloor spaces that are impractical to access are not required to have insulation). The RTA also requires landlords to comply with applicable requirements of other enactments in respect of buildings, health, and safety, meaning tenants can in theory use the RTA to enforce the HIR. However, RTA requirements are only enforced against landlords if tenants are successful in lodging a complaint with the Tenancy Tribunal.

Under the 2016 amendments to the RTA, the Ministry of Business, Employment, and Innovation (MBIE) may take cases against landlords on behalf of tenants. By its third month in operation, May 2017, the MBIE Tenancy Compliance and Investigations team had taken one action under these provisions (on behalf of a family living in an unconsented garage). It is likely that MBIE action will be limited to extreme cases such as these rather than other housing problems.

NZ’s regulatory framework is closer to that of the UK than the US, both of which are discussed below. However, NZ regional and local body approaches to housing standards are uniformly minimal, while central government standards are only marginally higher, and proactive central government rental housing quality enforcement is in its infancy.

## 3. The UK Housing Health and Safety Rating System

The idea for a NZ housing minimum standard owes its beginnings to collaboration with developers of the UK HHSRS. The HHSRS is a health-based risk assessment methodology for the evaluation of housing conditions [21]. It has shifted the focus of housing quality assessment from identifying defects and deficiencies in the structure and facilities to the potential threat to health and/or safety attributable to the conditions—the effects of defects.

It was developed over 10 years by the University of Warwick Law School supported by the UK Building Research Establishment and the London School of Hygiene and Tropical Medicine. The work started with a UK government–commissioned review into legal controls on housing standards and expanded to include matching data on housing conditions (from the House Condition Survey) with data from Hospital Episode Statistics, mortality records, Accident & Emergency Departments, and general practice doctors’ visits [22]. 

The development included an extensive literature review of the evidence of links between the health and safety of occupiers and housing conditions, including peer-reviewed and grey literature from medical, architectural, engineering, and building related sources. From this review, 29 potential housing hazards were identified, each to a greater or lesser extent attributable to the condition of the dwelling.

The underlying principle adopted for the HHSRS was that “Any residential premises should provide a safe and healthy environment for any potential occupier or visitor.” To satisfy this principle, a dwelling should be designed, constructed, and maintained with non-hazardous materials. Where present, unavoidable potential hazards—such as gas, electricity, cooking facilities, stairs, windows, and doors—should be made as safe as possible.

HHSRS potential housing hazards differ widely. To allow for comparison between the hazards, the differing health outcomes were grouped into four “Classes of Harm” based on the degree of incapacity caused [23].

To compare the threat posed by hazards, a formula was devised to generate a numerical score to reflect the surveyor’s judgment of the conditions—the higher the score, the greater the potential risk. Scores are aggregated into bands from A to J, Band J being the safest possible, and Band A the most dangerous. Enforcement options vary depending on the Band achieved.

In 2006, after 10 years of development and trials, the HHSRS was incorporated into legislation as the prescribed statutory standard for assessing housing conditions in England and Wales, replacing the Standard of Fitness (originally introduced in 1954). Since then, its daily use by English and Welsh local authority officers has validated it and shown it to be a robust and practical tool. It has also been shown to be transferable, having been adopted in 2010 by the US Department for Housing and Urban Development as an option for “healthy homes grant” applicants to measure housing conditions [24]. While some local authorities carry out routine inspections, others mainly respond to complaints. Official Information Requests to local authorities indicate that the number of complaints about housing conditions to local authorities is far greater than the number of inspections carried out. This situation may mean that local authorities do not have the resources to carry out all the work required. In addition, relying on occupant complaints to initiate inspections means that some of the worst housing may never undergo inspection, as occupants may be unaware of their rights or afraid of asserting them [25].

Local housing authorities use the HHSRS to determine whether enforcement action is necessary under Part 1 of the UK’s Housing Act 2004. Enforcement options range from advice on hazards, through to Emergency Prohibition or Remedial Action orders, depending on the degree and type of hazards.

## 4. US Housing Standards

The US has federal housing standards for publicly-owned housing and subsidised privately-owned housing. Otherwise, US standards are fragmented and vary by jurisdiction. Few of these housing standards are based on health criteria.

The 1936 Housing Act in the US contains general language about “decent, safe and sanitary” housing, but the Federal healthy housing program in the US formally began in 1999 with a relatively small grant programme. 

One of two main federal standards is required for private owners wishing to obtain a housing voucher subsidy: either the Housing Quality Standards, a pass/fail system, under which corrective measures are unsubsidized [26], or the Uniform Physical Condition Standard, which provides a numerical score, under which corrective measures may be subsidised. In each case, most standards are not related to health issues but are required to be independently inspected [27,28]. 

A few jurisdictions have adopted the National Healthy Housing Standard [29], an update to the American Public Health Association’s 1985 Basic Housing Inspection Manual. Other jurisdictions follow private sector “model codes”, which do not focus on health and safety and are usually only enforced following a major incident or a complaint by a tenant. 

Some cities have proactive inspections. Los Angeles, for example, inspects all rental housing every several years. Other cities, such as Rochester, New York, also require proactive compliance with lead safety and other requirements, including lead dust testing. The introduction of this law decreased the odds that a child with elevated blood lead levels lives in rental housing, demonstrating the potential for the physical testing of rental properties to improve the health of renters [30]. Finally, the National Healthy Home Training Center and Network offers a credential for Healthy Housing Inspections in an effort to bring a health focus into the work of housing and health code inspectors.

## 5. The NZ Rental Housing Warrant of Fitness (WoF)

In the early 2000s, the University of Otago’s He Kainga Oranga/Housing and Health Research Programme developed the Healthy Housing Index (HHI) as a research tool for measuring housing quality [31]. The HHI focused on measuring housing conditions considered to influence health and safety. It drew on elements in the National House Condition Surveys created by the Building Research Association of NZ (BRANZ) and hazards highlighted in the English and Welsh HHSRS [32]. It is a comprehensive building inspection checklist which has been used to identify health and injury hazards in the home for the purposes of a range of studies covering insulation, heating, and home injury hazards [2,3,5,33].

However, while the HHI met research needs well, the two to three hours required for inspection made it less practical for use as a broader rental quality inspection standard. At the same time, other NGOs were also considering housing checklists for purposes beyond health, such as sustainability or building longevity. He Kainga Oranga, the NZ Green Building Council, and the NZ Accident Compensation Corporation (ACC), with input from a wide range of agencies, collaborated to develop criteria that would cover areas with the strongest health and injury prevention evidence base. Early discussions included the possibility of a star-rating system, but ultimately a simple pass/fail approach was preferred on the basis that NZ housing needed to reach a baseline before aiming for higher quality, and that the more complex UK approach, depending on a highly trained workforce to quantify health and safety risk, was unlikely to work in NZ, where no such workforce is established. The development of the WoF is further described in Gillespie-Bennett 2013 [34]. 

The draft WoF had 31 criteria covering heating, ventilation, insulation, structural stability, sanitation, and injury hazards. The checklist contained 63 items and was field-tested in 2014 across a range of housing types and climates in the cities of Auckland, Tauranga, Wellington, Christchurch, and Dunedin. The development and outcomes of that field test are described in Bennett el al. 2014 [35]. After field testing, two items were removed, and some others amended, as described in the June 2014 Housing Warrant of Fitness (WoF) Assessment Manual Version 2.1 [36], leaving the 29 point Rental Housing Warrant of Fitness listed in Appendix A.

WoF items were selected to meet the following criteria:

- Demonstrable, evidence-based health gain or injury prevention;
- A problem that a tenant was not permitted to fix without the landlord’s permission, or it was impractical for a tenant to fix; and
- Inspection could be carried out by a generalist (e.g., a public health officer or trained builder) with at least a year’s experience in building inspections, rather than a specialist tradesperson (e.g., an electrician or plumber).

## 6. From Assessment Tool to Public Policy: The Rental WoF Implementation Study

With the rental WoF field-tested, the next stage in rental housing quality policy development is applying the rental WoF more broadly in real-world conditions. Consultation with government agencies identified central government concerns over potential adverse unintended consequences of a national introduction of a mandatory standard. We therefore designed and had funded by the NZ Health Research Council a policy experiment to independently measure the social, economic (including rental market and council transaction costs), and health and safety effects of introducing the rental WoF at local body level. Local authorities for the cities of Dunedin and Wellington, which had been part of the rental WoF field test, volunteered to work with He Kainga Oranga to introduce the rental WoFs in their areas.

Two early study decisions were to focus only on rental properties and to aim for mandatory inspections for all properties. We chose to examine the effects of introducing a WoF for rental housing rather than all properties for two reasons. First, there is good evidence that rental properties are on average in worse condition than owner-occupied properties. Second, bringing properties that fail rental WoF requirements up to standard involves work requiring landlord permission under the RTA. Tenants are therefore in a different position to home-owners, as they cannot themselves choose to make any necessary changes to meet the rental WoF; and as a group, as discussed earlier, they rarely have the economic power to induce quality improvements through market forces.

We are aiming to measure the effect of mandatory or semi-mandatory independent inspections for two reasons. Previous research has shown, first, that landlords have a poor response to incentives [37,38], with incentive uptake skewed toward properties in higher socio-economic areas. Consequently, voluntary schemes have little impact on the poor quality of rental properties in areas where the renting population is already bearing the brunt of other socio-economic determinants of health. The second factor is that both home-owners and landlords overestimate the quality of their properties [39], meaning landlords cannot be relied on to accurately assess whether or not their properties meet WoF criteria.

### 6.1. Study Design

The study was designed as a non-random-controlled quasi-experiment involving two intervention local authorities and two matched controls.

### 6.2. Study Questions

Our broad study question is whether a rental WoF can be introduced by local authorities in a way that is practical for use by public and private landlords and tenants without having an adverse impact on rental supply or affordability, or local authority budgets, and still deliver health and social benefits to tenants and the city as a whole.

### 6.3. The Intervention Cities

Dunedin and Wellington Councils are to implement the rental WoF in their regions. Wellington is NZ’s capital city, located at the bottom of the North Island at 41.3° South and home to roughly 200,000 people. It has a temperate maritime climate with high winds (average speed 29 km/h), and high rainfall (1244 mm/year), but also high sunlight hours (2001–2250 h per year [40]), but a very hilly topography, leaving many houses in shadow for large parts of the day and houses on hilltops exposed to extreme wind gusts. It has NZ’s highest per capita income and highest education level; 41% of Wellington properties are in rental tenure (total households not owned and not held in a family trust” divided by “total households stated”) [41], housing 42% of the city population (where tenure is known). 

Dunedin has a (cooler) temperate maritime climate. It is located in the lower part of the South Island at latitude 45.9° South and has a population of 125,000; 32% of Dunedin properties are in rental tenure, housing 39% of the city population. Like Wellington, it is hilly. As a predominantly “university town”, its renting population is younger than average.

### 6.4. The Control Cities

Wellington’s control city is Lower Hutt. Lower Hutt is located close to Wellington at latitude 41.2° South, with a population of 102,000; many people working in Wellington commute from Lower Hutt. The average income in Lower Hutt is lower than in Wellington. 34% of Lower Hutt properties are in rental tenure, housing 38% of the city population.

Dunedin’s control city is Invercargill. Invercargill is further south than Dunedin, at latitude 46.4^o^ South, and has a smaller population of 52,000. Invercargill is also home to a relatively large student population, though a lower proportion than in Dunedin. 30% of Invercargill properties are in rental tenure, housing 33% of the city population.

**Figure 1:**
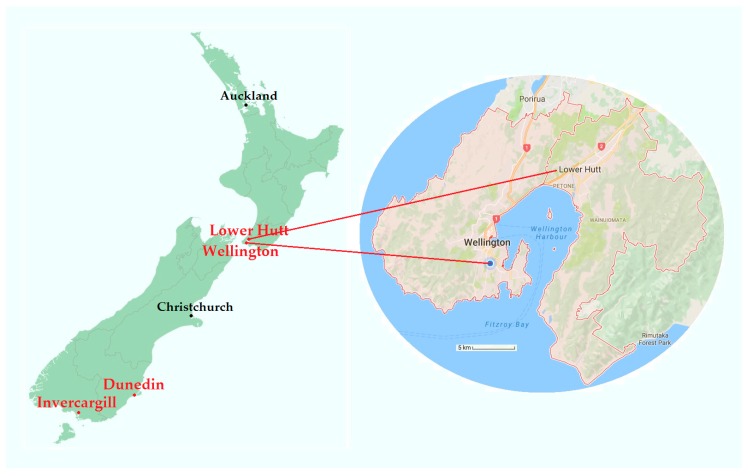
Locations of Wellington, Lower Hutt, Dunedin, and Invercargill. Map attribution: Ozhiker, and Google Maps.

Control cities have percentages of public housing similar to those of their matched intervention cities [42]. Intervention and control cities are mapped in Figure 1.

### 6.5. The Intervention

The intervention was intended to involve a mandatory rental WoF for all rental properties in the “treatment” cities. Our original regulatory options were either a “Local Bill” (a government statute available to address an issue particular to a region) or a local authority bylaw. Legal advice indicated that a local bill would likely be rejected, as poor rental housing quality was not particular to Wellington or Dunedin, and that local authorities did not have the power to require inspections necessary to a bylaw-based scheme. 

Our current proposed option is a hedonic pricing model involving local body property taxes (“rates”). Rates in Wellington and Dunedin are set according to property category (residential, commercial, industrial, with sub-categories of each) and property value. We have proposed councils separate residential-rated properties into two groups: owner-occupied properties and rented properties. Rates for rented properties would be set higher than rates for owner-occupied properties, with the additional revenue collected used to fund tenant advocacy and additional public health officers to carry out housing inspections. However, landlords could opt to have their properties independently inspected, and if their properties passed the rental WoF, their rates would be rebated to the same level as owner-occupied properties. The acceptance of this option is yet to be confirmed by either council.

While it decides how best to implement standards for rental housing, Wellington City Council is adopting an interim voluntary rental WoF scheme where landlords are able to request rental WoF inspections and advertise a rental WoF pass in marketing the property to rent. Landlords are encouraged to carry out their own pre-inspection check using the “Rental Housing WoF” app available free from Google Play and the iTunes App Store (the US HUD has also used apps to support its healthy homes programme, the “Healthy Homes Partners” app and the “Healthy Homes Basics” app, both available in the iTunes App Store). This interim stage provides landlords with an additional opportunity to meet minimum standards without a mandatory or quasi-mandatory inspection scheme.

### 6.6. Outcomes

Study question outcomes fall into four categories: social, economic, health, and policy. Outcomes will be compared between treatment and control cities, before and after introduction of the rental WoF.

#### 6.6.1. Social Outcomes

The primary social outcome of interest is residential mobility. Previous studies have found residential mobility to reduce social connectedness and community cohesion [43] as well as educational outcomes [44]. Tenant mobility can also increase landlords’ costs from advertising, time showing the property to prospective tenants, and lost rent between tenants. In NZ, the average length of tenure is 11 months in private rentals and three years in social housing, but only 6–7 months in the lowest private rental quartile. Schools in deprived areas report higher transience than schools in wealthier areas [45]. While economic pressures, family size changes, landlord-tenant relationship breakdown, and property sales all contribute to residential mobility, it is possible that tenants also seek new accommodation in the hope of trading up in quality. We therefore aim to measure whether the introduction of a rental WoF would reduce residential mobility, which will be measured using length of tenure from anonymised rental deposit (“Tenancy bond”) data in Statistics NZ’s Integrated Data Infrastructure (IDI) [46]. 

Mobility outcomes will be disaggregated by sector of tenure to identify whether the rental WoF can reduce what social housing providers describe as the “rebound” effect, where tenants who have left social housing for the private rental sector later reapply for social housing, because of the poor quality of the private rental sector. 

#### 6.6.2. Economic Outcomes

Some, including the current government and landlord advocates, have argued that requiring rentals to meet a rental WoF would impose high additional costs on landlords and that either these costs would be passed on to tenants as higher rents or landlords would exit the market, reducing rental housing supply, which would also cause rents to increase [47,48,49]. Initial analysis suggests that the impact of WoF compliance on rents would in large part depend on the mode of enforcement [50,51]. A literature review of the possible economic outcomes of introducing a rental WoF, based mainly on studies looking at the introduction of energy efficiency standards, found that this requirement is likely to increase rents, but this effect is likely to depend on market conditions: rents will not rise if tenants are unable to pay more [52]. 

We aim to examine the effects of a rental WoF on rental housing supply and affordability. NZ’s largest online rental listing company, Trademe, has agreed to provide data on rental listings in intervention and control cities over the 2016–2019 study period. The data to be provided for each property listed include the small area census unit (meshblock), the number of bedrooms, the advertised rent, date of listing, and date listing withdrawn. We aim to compare changes in the availability and cost of rental properties between suburbs matched for socio-economic status in intervention and control cities.

Finally, one of NZ’s largest home insurance companies has agreed to provide claims data for rental properties, which will allow us to assess whether improving the basic standard of rental properties reduces insurance claims.

#### 6.6.3. Health Outcomes

NZ has good quality nationwide health data collections. Health outcomes to be measured in the study are changes in pharmaceutical prescriptions, hospital admissions, and mortality rates, national accident insurance (ACC) claims for housing-quality-related accidents in the home, and aggregate school absentee data.

### 6.7. Analysis

We aim to measure differences in social, economic, and health outcomes between treatment and control cities before and after intervention, controlling for demographic differences between treatment and control cities. The effect can be described by the following equation:

(1) $$E^{O}=\frac{C^{i}}{C^{c}}=\frac{B^{i}-A^{i}}{B^{c}-A^{c}}$$

where *E* = effect, *O* = outcome, *C* = change, *B* = before, *A* = after, *i* = intervention city (Wellington or Dunedin), *c* = control city (Lower Hutt or Invercargill).

### 6.8. Limitations

This study relies on local authorities to volunteer, so it is not random. We assume policy implementation is generally more likely to be successful, and the local authority more likely to resolve barriers, when the local authority supports the policy. Implementation might face different barriers were local authority implementation required by central government.

The timeframe for measuring effects of implementation is also shorter than ideal, but is limited by funding and a scheduled 2019 increase in rental housing insulation standards, which will dilute the rental housing quality difference between treatment and control cities. The short “after” period is expected to underestimate health effects because under a mandatory scheme, the WoF would only be required when the property is re-let or within three years, and we estimate only half of rentals would need to meet mandatory standards within the study timeframe. 

It is unclear whether economic effects will be underestimated or overestimated. Rents and rental supply may take time to catch up to the new requirements or may show an initial sharp change that later returns to trend, as deferred below-market rent increases are updated to recoup costs, rental properties are temporarily taken out of the market to carry out deferred maintenance, and/or budget-sensitive landlords are prompted to sell up.

## 7. Conclusions

In this paper, we have summarised the origins and development of the NZ rental WoF, including knowledge gained from rental housing quality standards in the UK and the USA. We then described the method to be used in a non-random-controlled quasi-experimental study of the introduction of the rental WoF at local body level in NZ as a rental housing quality intervention. This study is designed to test whether the rental WoF is practical to introduce on a broad scale and whether it can improve rental housing quality without adverse consequences for tenants. It is expected to conclude in 2019. If the study results in positive outcomes for health without negatively affecting rents or rental supply, these findings will present a strong case for introducing a rental WoF nationwide as an effective public health intervention. 

Potential next steps will depend on the political landscape and involve promoting central government policy change to improve rental housing quality requirements with greater proactive enforcement, taking lessons from approaches found to be successful in the Rental WoF study and/or working with other Councils for policy change at a local body level. The UK and US examples show that both central and regional housing quality standards can bring improved housing quality for tenants, particularly with adequate enforcement and, potentially, subsidies for housing improvements. The Rental WoF study is a unique experiment designed to test policy efficacy and outcomes before broader introduction.

## Appendix A. WoF Assessment Criteria (See www.healthyhousing.org.nz for More Details)

1. Is there a functional, safe stove-top and oven? (Yes/no)
2. Is there adequate space for food preparation and storage? (Yes/no)
3. Is there an adequate supply of hot and cold potable water? (Yes/no)
4. Is the hot-water at the tap 55 °C (±5 °C)? (Yes/no)
5. Is there a functional toilet, which does not have a cracked or broken seat, cistern or bowl? (Yes/no)
6. Is there a suitably located bath or shower in good working order? (Yes/no)
7. Are there secure or high level cupboards or shelves for storing hazardous or toxic substances out of children’s reach? (Yes/no)
8. Is there an adequate form of safe and effective space heating? (Yes/no)
9. Do the bathroom, kitchen and all bedrooms have some form of ventilation to outside? (Yes/no)
10. Is the house reasonably free of visible mould, i.e., the total area of mould is less than an A4 sheet of paper? (Yes/no)
11. Are power outlets, light switches and wiring safe and in good working order? (Yes/no)
12. Is there adequate indoor lighting? (Yes/no)
13. Does the house have adequate working smoke alarms? (Yes/no)
14. Have the windows got effective latches? (Yes/no)
15. Do high level windows have security stays to prevent falls? (Yes/no)
16. Are there curtains or blinds in the bedrooms and living area? (Yes/no)
17. Do glass doors have safety visibility strips? (Yes/no)
18. Does the house have ceiling insulation to WoF standards? (Yes/no)
19. Does the house have underfloor insulation to WoF standards? (Yes/no)
20. Is a ground vapour barrier installed under the ground floor? (Yes/no)
21. Is the house weathertight with no evident leaks, or moisture stains on the walls or ceiling? (Yes/no)
22. Is the house in a reasonable state of repair? (Yes/no)
23. Is the storm and waste water drainage being adequately discharged? (Yes/no)
24. Is there any water ponding under the house? (Yes/no)
25. Is there adequate outdoor lighting near entrance ways? (Yes/no)
26. Does the house appear to be structurally sound? (Yes/no)
27. Are there handrails for all internal stairs and all outdoor steps that access the house, and do balconies/decks have balustrades to the current Building Code? (Yes/no)
28. Is the address clearly labelled and identifiable? (Yes/no)
29. Are there securely locking doors? (Yes/no)
